# Initiating human articular chondrocyte re-differentiation in a 3D system after 2D expansion

**DOI:** 10.1007/s10856-017-5968-6

**Published:** 2017-09-05

**Authors:** Abhijith K. Kudva, Frank P. Luyten, Jennifer Patterson

**Affiliations:** 1 Department of Materials Engineering, KU Leuven, Kasteelpark Arenberg 44, P.O. box 2450, Leuven, 3001 Belgium; 2 Skeletal Biology and Engineering Research Center, KU Leuven, O&N 1, Herestraat 49, P.O. box 813, Leuven, 3000 Belgium; 3 Prometheus, Division of Skeletal Tissue Engineering, KU Leuven, O&N 1, Herestraat 49, P.O. box 813, Leuven, 3000 Belgium

## Abstract

**Abstract:**

Cartilage damage affects a large population via acute and chronic injury and disease. Since native cartilage does not self-renew, cartilage tissue engineering has gained traction as a potential treatment. However, a limiting factor is that the primary cell type in cartilage, the articular chondrocyte, tends to de-differentiate when grown on 2D surfaces for in vitro expansion. Thus, 3D systems are being developed and used to counter this loss of chondrogenic capabilities. We hypothesize that a 3D matrix that can be remodeled may be more supportive of the chondrogenic phenotype of encapsulated articular chondrocytes than a 2D surface and may allow for the re-differentiation of chondrocytes after 2D expansion. Hence, in this study, enzymatically degradable polyethylene glycol (PEG) hydrogels containing two different protease degradable peptide segments, with different degradation rates, were tested in combination with chondrogenic medium as a 3D in vitro culture system to better recapitulate the native environment of human articular chondrocytes (hACs). In addition, the effect of incorporation of the integrin binding ligand Arg-Gly-Asp (RGD) in the hydrogels was explored. Hydrogels crosslinked with a slower degrading crosslinker and not functionalized with RGD maintained hAC viability and led to increased GAG production and chondrogenic gene expression over time, suggesting that this system can initiate hAC re-differentiation after 2D expansion.

**Graphical abstract:**

## Introduction

### Reflection on career goals by Jennifer Patterson

**Fig. 1:**
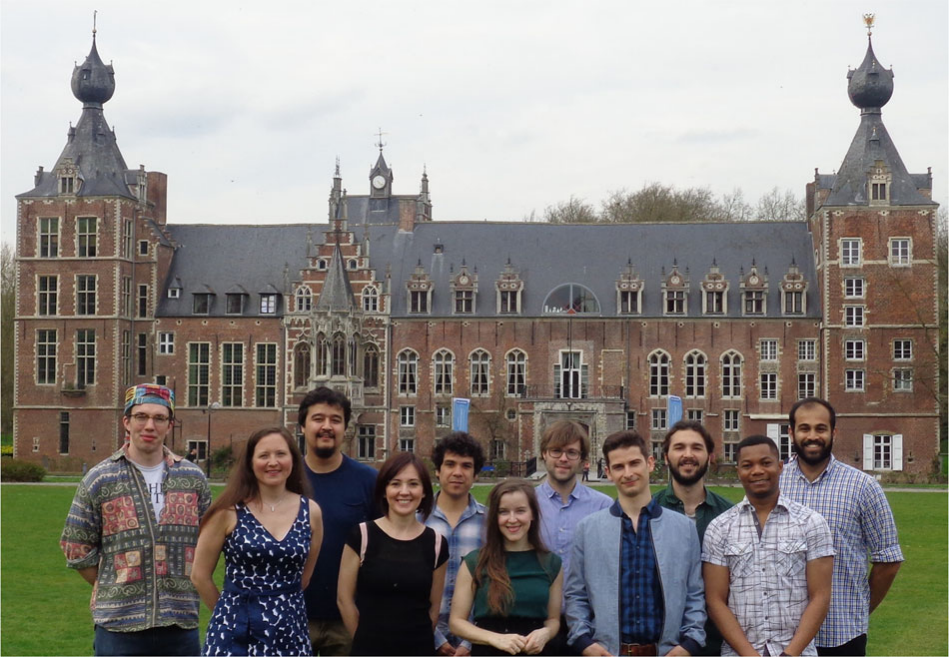
Research Group of Jennifer Patterson (March 2017, in front of the Arenberg Castle on the engineering campus of the KU Leuven): Front row: Dr. Jennifer Patterson, Dr. Susanna Piluso, Laurien Van den Broeck, Jasper Timmerman, Dr. Soultan Al Halifa; Back row: Rory Gibney, Burak Toprakhisar, Christian Garcia, Laurens Rutgeerts, Ricardo Augusto, Abhijith Kudva

While studying chemical engineering at Princeton University, I participated in a Research Experience for Undergraduates program at MIT in the laboratory of Prof. Jonathan King and did senior thesis research with Prof. Michael Hecht, where I determined the morphology of novel beta-sheet proteins. Afterwards, I worked in a start-up company founded by Profs. Michael Cima and Linda Griffiths to develop 3D printing for tissue engineering. As I wanted an academic career, I went to the University of Washington for a PhD in bioengineering, which was supported by a NSF Graduate Research Fellowship, and worked with Prof. Patrick Stayton on hyaluronic acid hydrogels. During my studies, I read papers by Prof. Jeffrey Hubbell that described modular PEG-peptide hydrogels and decided that I wanted to work with them so I obtained a postdoctoral fellowship from the Whitaker International Program to go to the Hubbell lab at EPFL. In 2011, I started as a tenure-track assistant professor in the Department of Materials Engineering at KU Leuven to develop a research line on materials-biology interface science. My group grew to comprise two postdocs, six PhD students, and two master students (Fig. 1) and was supported by four major project grants and several individual fellowships totaling over €2 million. I also developed master-level courses on the host response and ‘next generation’ biomaterials. In fall 2017 I am starting a new position as CSO of BIOFABICS LDA, a start-up company specialized in 3D biotissue analogues, but hope to return to academia in the future.

### Background and state of the art

Articular cartilage, also known as hyaline cartilage, is the main load bearing tissue at synovial joints. To be able to bear the load exerted on it and maintain its mechanical durability, articular cartilage is comprised of a dense extracellular matrix (ECM) that is primarily composed of type II collagen, proteoglycans, and other noncollagenous proteins, which are present to a lesser extent [1, 2]. These components interact with each other to create a network that helps entrap large amounts of water, which is vital for the maintenance of the biomechanical functionality of the tissue [3]. Although it is highly durable, articular cartilage, just like other components of the body, is susceptible to damage via everyday wear and tear, traumatic injury, and/or disease. But, unlike other tissues, articular cartilage is avascular, alymphatic, and aneural, and it contains a very sparse, specialized cell population called chondrocytes. These properties inhibit native cartilage from renewing itself when damaged, leading to chronic pain, discomfort, and lowered quality of life for patients. Therapies in the past, such as alloplastic or allogenic implants, were successful to a certain extent but were limited by donor tissue availability and donor site morbidity. More recently, surgical techniques such as microfracture surgery or autologous chondrocyte transplantation/implantation (ACT/ACI), although promising [4, 5], are marred by the size of the damage, fibrous cartilage formation with inferior mechanical properties, and donor site morbidity [6]. Moreover, a limiting factor when using ACT/ACI is that the harvested chondrocytes de-differentiate into a fibroblast-like cell type when expanded in vitro on two dimensional (2D) surfaces to desired cell numbers [7, 8]. To counteract this loss of chondrogenic state of the chondrocytes, researchers have been investigating several methods of culture in three dimensional (3D) systems. It was first shown using agarose gels that de-differentiated chondrocytes can re-differentiate and regain some of their phenotype under 3D conditions [8]. Since then, to develop various 3D re-differentiation techniques for articular chondrocytes, investigators have systematically moved from seeding the cells on meshes and scaffolds [9, 10] to embedding cells within constructs, primarily hydrogels.

Hydrogels are an attractive scaffold choice for cartilage damage repair as they are made up of large amounts of water, thus mimicking the native ECM where chondrocytes reside. Additionally, the hydrophilic nature of these scaffolds facilitates nutrient transfer throughout the system. Autologous chondrocytes combined with natural polymer based hydrogels such as agarose [8, 11], alginate [12, 13], hyaluronic acid [14, 15], and fibrin [16, 17] have garnered attention in the past due to their attractive in vivo/in situ application and have subsequently demonstrated sufficient glycosaminoglycan (GAG) content and histological evidence of cartilaginous tissue. Although the aforementioned natural polymer hydrogels have shown promising results, controlling the mechanical properties and structural integrity of these hydrogels while in culture in vitro and the lack of uniformity of these hydrogels are a few of the limitations that arise with these types of systems. In particular, in vitro stability is a desired feature, thus making a synthetic material, such as polyethylene glycol (PEG), an attractive alternative. PEG, although inert, can be combined with natural components such as matrix metalloproteinase (MMP) sensitive peptide linkages or other bioactive ligands, and these systems have demonstrated increased bioactivity and proved advantageous for tissue engineering purposes [18]. Hydrogels formed from PEG macromers have been extensively studied and characterized, and the different compositions that allow for the controlled variation of properties such as stiffness, degradation rate, and bioactivity have been thoroughly demonstrated [19, 20]. Moreover, chondrocytes encapsulated within PEG-based constructs have displayed promising results in the past. For instance, when bovine chondrocytes were photo-encapsulated within PEG dimethacrylate (PEG-DM) 3D systems, they showed a propensity to produce collagen II and new ECM [20, 21]. Additionally, it was shown that the incorporation of degradable linkages, in this case hydrolytically sensitive poly(lactic acid) (PLA) segments, increased the extent of collagen II production and the homogeneity of GAG deposition by encapsulated bovine chondrocytes [20]. In similar manner, researchers have shown that combining a collagen mimetic peptide conjugate with a photo-polymerized PEG system enhances chondrocyte ECM production [22].

Hence, herein, a chemically crosslinked, protease sensitive PEG vinyl sulfone (PEG-VS) system [18] was utilized to encapsulate human articular chondrocytes (hACs), as a 3D system for the in vitro culture of hACs. In the past, MMP sensitive synthetic polymer systems have been investigated with primary bovine (calf) chondrocytes [23]. This study tested two different sized PEG-VS macromers and further compared a MMP sensitive and a non-degradable crosslinker. All of the systems maintained bovine chondrocyte viability over time; however, the MMP sensitive structures led to an increased expression of collagen type II and aggrecan [23]. Building upon these results, in the present study, the use of hACs improves the translational value of a MMP sensitive PEG matrix for future cartilage tissue engineering applications, an aspect that is vital to the field of regenerative medicine. Furthermore, two protease-degradable peptide segments with different kinetic parameters [24] were tested in combination with and without the integrin binding sequence, Arginine-Glycine-Aspartic Acid (RGD), in a PEG hydrogel system for the purpose of supporting the re-differentiation process of de-differentiated chondrocytes that were previously expanded in 2D culture.

## Materials and methods

### Articular chondrocyte cell culture

Frozen vials of hACs at passage 2 were acquired from the Tissue Homeostasis and Disease (THD) Lab, part of the Skeletal Biology and Engineering (SBE) Research Center, at KU Leuven. The cells were isolated from the hips of patients undergoing total hip replacement surgery. The University Hospitals Leuven Ethics Committee and Biobank Committee approved the study, and specimens were taken with patients’ written consent. hACs were plated in tissue culture flasks with regular growth medium (GM) (DMEM/F12 with 10% fetal bovine serum (FBS), 1% antibiotics (AB), and 1% L-glutamine) and used at passage 5 for encapsulation within the 3D hydrogels.

### Hydrogel preparation and cell encapsulation

6.5% (w/v) cell-laden PEG-vinyl sulfone (PEG-VS) hydrogels were made via a Michael Type addition reaction as previously described [18]. Briefly, 20 kDa 4-arm PEG-VS (JenKem) was dissolved in 0.3 M HEPES buffer, pH 8, for a final w/v percentage of 6.5%. Subsequently, the adhesion binding peptide containing a free thiol (Ac-G**C**GYGRGDSPG-NH_2_) was reacted with the PEG-VS for a final concentration of 150 µM within the 3D scaffold. With regards to the hydrogels without RGD (0 µM), an equal volume of buffer was added. Next, a trypsin released hAC cell suspension (10 × 10^6^ cells/ml final concentration) was mixed with two different di-thiol, MMP sensitive crosslinkers with different degradation rates [24]: Ac-G**C**REGPQGIWGQER**C**G-NH_2_, referred to as the regular degrading crosslinker (R), or Ac-G**C**RDVPMSMRGGDR**C**G-NH_2_, referred to as the fast degrading crosslinker (F). Finally, the PEG-VS solutions, with or without RGD functionalization, were reacted with their corresponding crosslinker and cell suspension. The hydrogel nomenclature used throughout the remaining text is given in Table 1. Hydrogel disks of approximately 1 mm in thickness and about 8 mm in diameter were formed by sandwiching drops of the hydrogel precursors between hydrophobic SigmaCote (Sigma) treated slides with spacers and incubated for 30 min at 37 °C in a humidified incubator. Peptides were purchased from commercial suppliers (Biomatik or PeptideSynthetics) or were prepared by solid phase peptide synthesis and purified via HPLC-MS.

**Table 1:** Hydrogel nomenclature

Hydrogel abbreviation	Hydrogel composition
R0	6.5% PEG + Regular degrading crosslinker + 0 μM RGD
RR	6.5% PEG + Regular degrading crosslinker + 150 μM RGD
F0	6.5% PEG + Fast degrading crosslinker + 0 μM RGD
FR	6.5% PEG + Fast degrading crosslinker + 150 μM RGD

### Culture conditions

**Fig. 2:**
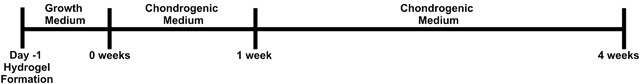
Schematic representation showing the culture conditions and experimental time points

Cell-laden hydrogels were carefully removed from the slides and placed in 24-well plates with regular GM for 24 h and then switched to 4 C chondrogenic differentiation medium [DMEM/F12 (Invitrogen) with 5% FBS, 1% AB, 10 ng/ml TGF-β1 (PeproTech), 1× Insulin Transferin Selenium + premix (ITS + ; BD Biosciences), 100 nM dexamethasone, and 100 µg/ml ascorbic acid] [25, 26], which was replenished every 2–3 days. Samples were analyzed at 0 weeks (immediately before switching to chondrogenic differentiation medium) and after 1 week and 4 weeks in the chondrogenic differentiation medium (Fig. 2).

### Picogreen/QuanIT DNA quantification

To investigate cell proliferation, the DNA content of three hydrogels per condition and per time point was calculated using the Picogreen DNA Quant-iT kit and the Qubit measuring device (Invitrogen). The hydrogels were first degraded in digestion buffer composed of Proteinase-K (0.5 mg/ml; Sigma Aldrich), phosphate buffered EDTA (5 mM EDTA in PBS pH 7.1; PBE), and 0.1% Triton X-100 solution overnight at 60 °C. Next, a working solution of the reagent was prepared according to the instructions of the manufacturer. The samples were read using the Qubit Fluorometer, and the DNA concentration was calculated.

### Cell viability assay

The viability of the encapsulated hACs within the different PEG hydrogel constructs was analyzed using a Live/Dead kit (Invitrogen). The hydrogels were washed with phosphate buffered saline (PBS; Gibco) for 5–10 min. Next, a staining solution of calcein AM and ethidium homodimer-1 (ETH-1) in PBS was added to the wells and incubated at 37 °C for 30–45 min. The samples were then washed again in PBS and imaged using an Olympus FluoView confocal microscope with 10× objective at step size of 10 μm and a total thickness of 500 μm. Imaris software was used to quantify the images in the 3D view, and all images are represented as the maximum intensity of the z-stack.

### DMMB GAG assay

The GAG production was analyzed using the dimethylmethylene blue (DMMB) GAG assay. At each time point, the same digested samples used in the DNA assay were used for the DMMB assay to quantify GAG/DNA. Briefly, 1,9–dimethylmethylene blue chloride (Sigma) was dissolved in ethanol overnight and then added to a sodium chloride (0.04 M NaCl)/glycine solution, pH 3, for a final concentration of 46 μM DMMB. The solution was filtered, and the absorbance at 525 nm was measured to be 0.314 OD. Next, a serial dilution of chondroitin sulfate (CS; Sigma), ranging from 0 to 100 µg/ml, was prepared using PBE. Finally, 30 μl of the samples or the CS standards was loaded into a 96-well plate, along with 270 μl of the DMMB dye solution, and the absorbance was read at 570 nm. The GAG concentration was calculated using the standard curve generated from the serial dilution of CS, and then divided by its corresponding DNA content to calculate GAG/DNA (µg/pg).

### RNA extraction and quantitative PCR for chondrogenic markers

The total RNA from the hydrogels was isolated using a trizol/ethanol/chloroform RNA extraction method and quantified using a NanoDrop ND-1000 spectrophotometer (Thermo Scientific). Subsequently, 500 ng of RNA was converted into complementary DNA (cDNA) with Oligo(dT) 18 primer using the RevertAid Hminus First Strand cDNA Kit (Fermentas). SYBR Green (Applied Biosystems) primers and a Rotor-Gene sequence detector were used for qPCR of chondrogenic gene markers such as *SOX-9*, aggrecan *(ACAN*), and type II collagen (*COL2A*). At each time point, each independent triplicate sample was measured in duplicate. Relative expression was calculated using the 2^-ΔCT^ method, normalized to the house keeping gene, hypoxanthine-guanine phosphoribosyltransferase-1 (*HPRT-1*).

### Statistical analysis

Quantitative results in all text and figures are expressed as mean ± standard deviation. For the DNA quantification, GAG production, and chondrogenic gene expression, a two-way ANOVA was performed using hydrogel composition and time as variables. Results were considered significant with p-values below 0.05 (*p < 0.05; **p < 0.01; ***p < 0.001).

## Results

### Hydrogel composition influences hAC proliferation but not viability over time

**Fig. 3:**
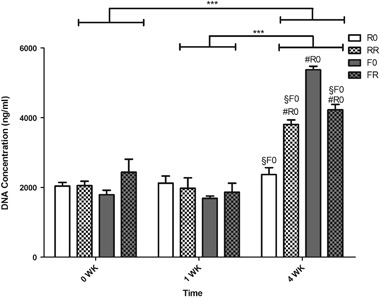
DNA quantification of encapsulated hACs within protease degradable PEG hydrogels, with and without RGD, and cultured over 4 weeks. Constructs were analyzed at 0 weeks (immediately before switching to chondrogenic medium) and after 1 week and 4 weeks in the 4 C chondrogenic differentiation medium. Results are presented as mean ± standard deviation (n = 3, *** p < 0.001; #R0 p < 0.001 when comparing to R0; §F0 p < 0.001 when comparing to F0)

The DNA content of the various constructs was analyzed to observe the effect of hydrogel composition on the proliferation of the encapsulated hACs. When looking at the DNA content (Fig. 3) of the hydrogels after 24 h of culture in GM, the hydrogels did not demonstrate a significant difference in DNA content, thus indicating non-biased cellular encapsulation. Furthermore, after being switched to the 4 C chondrogenic medium for 1 week, the DNA content of the hydrogels did not indicate a significant difference from week 0 nor was there a significant difference among the different hydrogel compositions. However, after 4 weeks of culture, the hAC cell numbers increased significantly (p < 0.001) in three out of the four hydrogel compositions, compared to both 0 and 1 weeks, indicating cellular proliferation within these formulations over time. Only the hydrogels composed of the regular crosslinker and not functionalized with RGD did not demonstrate a significant increase in DNA content over time. Morever, at the 4 week time point, this same hydrogel composition demonstrated a significantly lower amount of DNA not only when compared to its counterpart functionalized with the integrin binding sequence RGD (p < 0.001) but also when compared to both faster degrading hydrogel formulations (p < 0.001). Interestingly, the hydrogels with the faster degrading crosslinkers displayed a higher DNA content after 4 weeks of in vitro culture than the hydrogels composed of the regular degrading crosslinker. The hydrogels composed of the faster degrading crosslinker and not functionalized with RGD displayed the highest increase in DNA content over time as well as the highest amount of DNA content compared to the other formulations (p < 0.001).

**Fig. 4:**
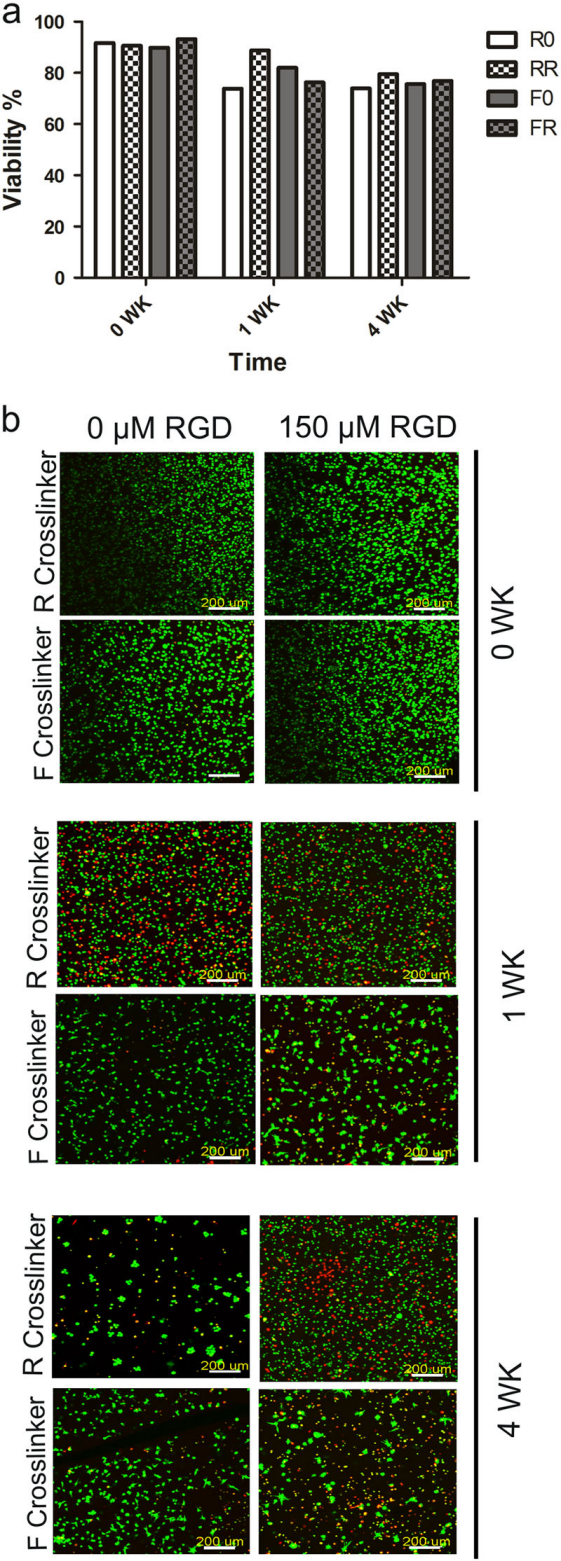
hAC viability within protease degradable PEG hydrogels, with and without RGD, cultured over 4 weeks. Constructs were analyzed at 0 weeks (immediately before switching to chondrogenic medium) and after 1 week and 4 weeks in the 4 C chondrogenic differentiation medium. **a** Quantification of the viability of hACs encapsulated in hydrogels using IMARIS software (n = 1). Values given are the average of three different areas measured within one sample. **b** Representative images of Live/Dead staining via calcein AM (green, live cells) and ethidium homodimer-1 staining (red, dead cells). Confocal images are depicted as the maximum intensity of a 500 µm z-stack that was acquired using a 10× objective (scale bar = 200 µm)

When observing the viability of the hACs in the 3D PEG hydrogels (Fig. 4), the cells were able to stay alive throughout the 4 weeks of the study. More specifically, after 24 h of culture in GM, the hACs maintained a viability of > 90% in all the hydrogels (Fig. 4a). Next, when looking at the cell-laden hydrogel constructs that were cultured in 4 C chondrogenic medium for one week, the hACs demonstrated a slight drop in viability to approximately 75% in the different hydrogel constructs. Additionally, the cells encapsulated in the RGD containing hydrogel constructs showed a slightly more spread morphology (Fig. 4b) compared to the hACs within hydrogels without RGD, which was expected due to the presence of the cell adhesive ligand. Lastly, after 4 weeks of in vitro culture in 4 C chondrogenic medium, the hACs continued to demonstrate a viability of ~70%, an indication that the selected protease-degradable PEG hydrogels are not harmful to the enapculated hACs over time.

### GAG production by hACs increases after 3D in vitro culture in chondrogenic medium

**Fig. 5:**
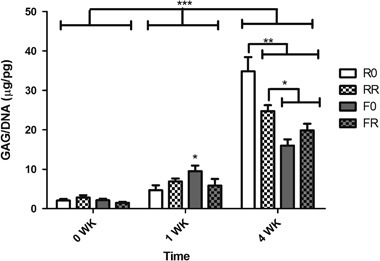
GAG production normalized to DNA content by hACs within protease degradable hydrogels, with and without RGD, cultured over 4 weeks. Constructs were analyzed at 0 weeks (immediately before switching to chondrogenic medium) and after 1 week and 4 weeks in the 4 C chondrogenic differentiation medium. Results are presented as mean ± standard deviation (n = 3, *p < 0.05, **p < 0.01, *** p < 0.001)

The re-differentiation of the encapsulated hACs was observed in the 3D PEG hydrogels via the increase in GAG production over time (Fig. 5). More specifically, the GAG content, normalized to DNA content, demonstrated a dramatic increase in all the hydrogels after in vitro culture in chondrogenic medium. When looking at the GAG production of the encapsulated hACs after 24 h in GM, there was little to no GAG present. Furthermore, at week 0, no significant difference among the different hydrogel groups was observed, as expected. Already at 1 week, there was a significant increase in GAG content compared to 0 weeks for all the groups (p < 0.001). Although the constructs did not demonstrate an increase in DNA content at this time point, the cells were producing more GAG when cultured in the chondrogenic conditions. Moreover, the hACs encapsulated within the hydrogel composed of the faster degrading crosslinker and no RGD produced significantly more GAG per cell compared to the cells within the other hydrogel groups (p < 0.05). Besides this, there was no other significant difference observed among the hydrogel compositions at 1 week. On the other hand, after 4 weeks of in vitro culture in chondrogenic medium, all the hydrogel compositions displayed a 5×–7× increase in new GAG production per cell by the hACs within, which was significantly higher than the GAG production at both 0 and 1 weeks (p < 0.001). This demonstrates the effect that 3D culturing systems have on GAG production by hACs. Additionally, at 4 weeks, the trend in GAG production is the opposite of that for the DNA content of the constructs. The hydrogels comprised of the regular degrading crosslinker seem to allow the encapsulated hACs to produce significantly more GAG per cell (p < 0.05) than the cells within the hydrogels with the faster degrading crosslinker. Specifically, the hACs in the hydrogels with the regular crosslinker that were not functionalized with RGD displayed the highest amount of GAG per DNA compared not only to the hACs in the regular degrading hydrogel with RGD but also to hACs in the faster degrading hydrogel formulations (p < 0.01). This was the same hydrogel composition that displayed the lowest DNA content.

### Recovery of hAC chondrogenic gene expression in a 3D environment

**Fig. 6:**
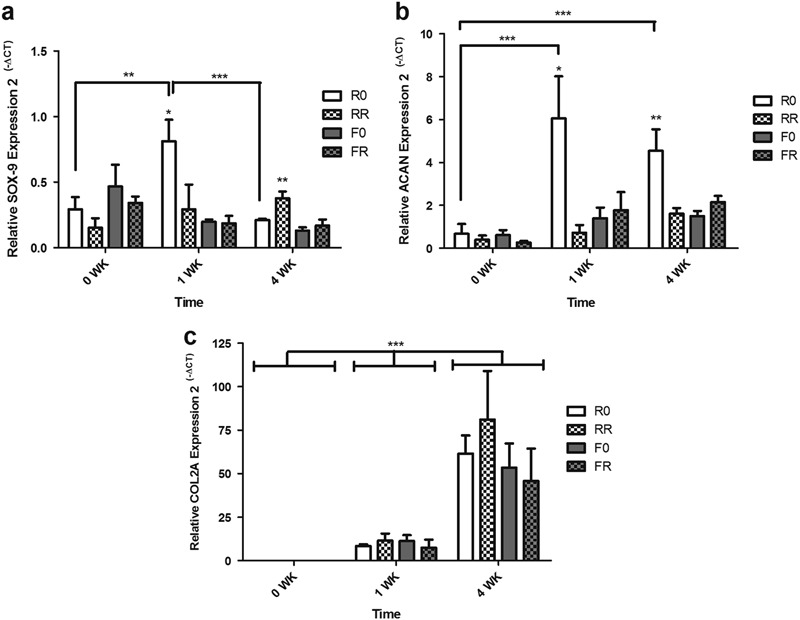
Chondrogenic gene expression of hACs within protease degradable hydrogels, with and without RGD, cultured over 4 weeks. Constructs were analyzed at 0 weeks (immediately before switching to chondrogenic medium) and after 1 week and 4 weeks in the 4 C chondrogenic differentiation medium. Gene expression was calculated relative to the housekeeping gene, HPRT. **a** *SOX-9*; **b** *ACAN*; **c** *COL2A*. Results are presented as mean ± standard deviation (n = 3, *p < 0.05, **p < 0.01, *** p < 0.001)

Lastly, the chondrogenic gene expression of the encapsulated hACs in the 3D hydrogels demonstrated a significant increase over time, an indication that re-differentiation was taking place (Fig. 6). *SOX-9* (Fig. 6a), an early marker for chondrogenesis, demonstrated no significant difference among the various hydrogel formulations at 0 weeks, in addition to relatively low expression levels. After 1 week of in vitro culture, the hACs cultured in the constructs with the regular crosslinker and with no RGD witnessed a significant upregulation in *SOX-9* expression levels compared to the hACs within the other hydrogels (p < 0.05). This was also significantly higher than at 0 weeks (p < 0.01). After 4 weeks of culture, the hACs displayed a drop in *SOX-9* expression levels within the hydrogels comprised of the regular crosslinker and no RGD compared to its 1 week counterpart (p < 0.001), while displaying relatively little change in the other hydrogel formulations. Moreover, at the 4 week time point, the hACs within the constructs with the regular crosslinker and functionalized with RGD displayed the highest expression of *SOX-9* compared to the cells cultured in the other compositions (p < 0.01). Additionally, the hACs demonstrated a general increase in *ACAN* (Fig. 6b) gene expression over time within the 3D constructs. The cells cultured in the hydrogels with the regular crosslinker and no RGD had a significant upregulation of *ACAN* gene expression already by 1 week (p < 0.001) and maintained this higher level of expression at 4 weeks (p < 0.001). Moreover, at 1 and 4 weeks, the cells cultured in these hydrogels displayed a significantly higher expression than the cells cultured in the rest of the hydrogel groups (p < 0.05 at 1 week, p < 0.01 at 4 weeks). Finally, the *COL2A* (Fig. 6c) gene expression levels of the encapsulated hACs further support the above data indicating re-differentiation taking place. There was a significant increase (p < 0.001) in relative gene expression of *COL2A* from 0 to 1 week of in vitro culture, but there was no difference observed among the different hydrogel formulations. This similar trend was observed at 4 weeks, where the *COL2A* expression by the hACs witnessed an even greater increase in all the hydrogel formulations (p < 0.001), but there was no significant difference observed among the various formulations.

## Discussion

With this study, we were able to demonstrate the use of an enzymatically degradable PEG hydrogel to maintain the viability and initiate the re-differentiation process of encapsulated de-differentiated hACs. Hydrogels containing the MMP sensitive, regular degrading crosslinker and not functionalized with RGD helped preserve hAC viability over 4 weeks but did not promote proliferation. Meanwhile, the hACs encapsulated in these same hydrogels exhibited higher GAG production and higher chondrogenic gene expression throughout the study. The recapitulation of the chondrogenic phenotype of de-differentiated chondrocytes in a 3D system after being cultured in 2D monolayers has been investigated in the past, most commonly in alginate beads or pellet cultures [27]. Moreover, it is well accepted that chondrocytes favor a 3D system over 2D in vitro culture as this more closely resembles their native environment [8, 28]. More specifically, the results gathered herein align well with previous research conducted on different PEG systems with both bovine [23] and human chondrocytes [29], where the particular 3D constructs used maintained chondrocyte viability and enhanced GAG production depending on the hydrogel composition.

While the hydrogels with the regular degrading crosslinker and without RGD performed the best here, all compositions of MMP sensitive PEG based hydrogels in this study maintained the viability of the encapsulated hACs over time. Additionally, the high viability observed at the 0 week time point indicates that the hACs remained viable when being incorporated into the 3D network and thus demonstrates that the hydrogel formation methodology in and of itself does not have any adverse effect on the viability of the hACs. In this way, the chemically crosslinked PEG hydrogels, which are formed at close to physiological pH [20], address a possible limitation of photo-crosslinked 3D systems [21], which often use cytotoxic photo-initiators and/or exposure to UV light.

The hACs, when embedded within the different hydrogels at a cellular density of 10 × 10^6^ cells/ml, did not demonstrate a significant increase in the DNA content until 4 weeks. This could be an indication that the encapsulated cells were still adjusting to their new 3D environment and thus not proliferating at the early time point, similar to what has been observed when hACs were cultured in alginate beads or in pellets [27]. The increase in DNA content at the later time point is also similar to that observed in studies where bovine articular chondrocytes photo-encapsulated in PEG hydrogels did not proliferate until after 4 weeks of culture time [19]. At the 4 week time point, the higher amount of DNA content in the hydrogels incorporating the faster degrading crosslinker is probably due to its faster degradation rate [24], which also correlates with previous studies using bovine chondrocytes in MMP sensitive, chemically crosslinked PEG systems [23] and in PEG co-block polymer based hydrogels [19, 20]. These studies showed not only that a degradable construct is favorable over a non-degradable one [23] but also that DNA content increased in constructs comprising a higher proportion of degradable components [19]. Interestingly, they also demonstrated that these same systems produced more GAG over time [19], a trend that was different within our investigations.

When looking at the GAG production, the low amounts of GAG at the 0 week time point indicate the de-differentiated state of the hACs due to the prior 2D culture. The hACs were released from the 2D flasks and used at passage 5 to ensure that hACs had indeed undergone de-differentiation and had gradually lost their characteristic molecular markers [30]. Although there was an increase in GAG content after 1 week, the biggest increase occurred after 4 weeks of culture. The correlation between the GAG/DNA content and the DNA results was different from what was observed in the above mentioned study by Bryant et al., which demonstrated that the faster degrading systems allowed the cells to proliferate more and produce more GAG over time [20]. Here, the constructs with the higher DNA content at 4 weeks, mainly the faster degrading hydrogels, displayed a lower amount of GAG production per cell, whereas the constructs with the regular degrading crosslinker demonstrated higher amounts of GAG. More specifically, cells encapsulated in the regular degrading hydrogel without RGD produced the highest amount of GAG/DNA. One possible explanation for this result could be due to the fact that the hACs prefer anchorage independent growth and re-differentiation [8] and thus, once placed within this environment, acclimated themselves and produced more GAG and laid down new ECM. On the other hand, the hydrogels containing RGD enabled the cells to have a spread morphology, thus inhibiting the chondrogenic process, a trend that was also seen when RGD was incorporated within agarose hydrogels [31].

Additionally, the overall low expression levels of the chondrogenic genes *SOX-9*, *ACAN*, and *COL2A* at 0 weeks and the general increase over time of these markers demonstrated that the hACs were in fact de-differentiated, which is consistent with reports of the 2D culture of these cells [32, 33], and that the cells were starting the re-differentiation process. Both studies demonstrated the loss of gene expression, especially for *ACAN* and *COL2A*, of serially passaged hACs [32, 33]. In the present study, *SOX-9* displayed a significant upregulation already at 1 week for cells in the regular degrading hydrogel without RGD as well as a drop in the expression at the 4 week time point. This fits with the status of *SOX-9* as an early marker for chondrogenesis, especially during cellular condensation and in differentiated chondrocytes [34, 35]. The significantly higher *ACAN* expression levels over time by the hACs in the regular degrading hydrogels without RGD further validated this as the hydrogel of preference for new GAG production. Taken together with the *SOX-9* expression and GAG/DNA data, these results suggest that the hACs may be undergoing the early stages of chondrogenesis at 1 week and are beginning to lay down their own matrix, especially in the regular degrading hydrogels without RGD. Lastly, the significant increase in *COL2A* expression from 0 to 1 to 4 weeks helps cement the idea that these enzymatically degradable PEG-VS hydrogel systems do indeed initiate the recovery/re-differentiation of hACs after they have undergone de-differentiation during 2D culture.

Chondrocytes, whether of animal or human origin, have been investigated in 3D hydrogels for an extended period of time. The enzymatically degradable PEG system used within this study, in particular the regular degrading hydrogel without RGD, is similar to the hydrogels used with bovine chondrocytes by Park et al. [23]. However, whereas they compared a degradable to a non-degradable PEG hydrogel with different elastic moduli depending on the size of PEG macromers used (4.5 and 13.5 kPa with a 4-arm PEG and an 8-arm PEG macromer, respectively) [23], in this study we tested two different proteolytically degradable peptides, with different k_cat_ values, incorporated within PEG hydrogels with or without RGD, thus adding an additional dimension. The regular degrading peptide crosslinker, Ac-G**C**REGPQGIWGQER**C**G-NH_2,_ had k_cat_ values of 0.65 ± 0.13 s^−1^ and 2.17 ± 0.16 s^−1^ with the enzymes MMP-1 and MMP-2, respectively [24]. The fast degrading peptide crosslinker, Ac-G**C**RDVPMSMRGGDR**C**G-NH_2_, had significantly higher k_cat_ values of 5.25 ± 0.95 s^−1^ and 4.82 ± 0.79 s^−1^ with the enzymes MMP-1 and MMP-2, respectively [24]. This difference in degradation rate of the peptide crosslinkers will also affect the relative mechanical properties of the hydrogels over time. While the starting hydrogels have similar storage moduli independent of the specific sequence of the peptide crosslinker (unpublished data), the difference in degradation rates will lead to hydrogels with different mechanical properties at later time points in the experiment. The differences that arise over time due to the two different crosslinkers in the hydrogel structures must be further investigated in future studies. Moreover, although different hydrogel formulations were preferred by the hACs for proliferation on the one hand and GAG production/gene expression on the other, in both cases the hydrogels did not contain RGD, further validating that hACs, once placed in a 3D structure, prefer a non-adherent environment. Although some of the results herein align themselves with prior studies, the inconsistencies can potentially be attributed to the fact the hydrogels used here and in the previous investigations are not composed of the exact same formulations. This further indicates the complexity of the factors that can influence chondrocyte phenotype, ECM synthesis, *etc*. In future work, a systematic design of experiments approach could help to elucidate the effects that the various factors have on chondrocyte in vitro culture.

## Conclusion

Overall, this study demonstrates that the selected protease-degradable, functionalized PEG hydrogels provide a suitable environment for the hACs to initiate re-differentiation and regain evidence of a chondrogenic phenotype. Although similar MMP-sensitive PEG hydrogels have demonstrated positive results when combined with bovine chondrocytes, herein we further highlight the system’s use as an in vitro system for hACs, taking it one step closer to translational/clinical relevance. The hydrogels that contained the regular degrading crosslinker and no RGD seemed to be the most appropriate scaffold for the hACs to recapture their chondrogenic capabilities in vitro. Although these hydrogels did not exhibit a significant increase in DNA content and thus failed to promote proliferation of hACs, they still helped to maintain the viability of the hACs, supported increased GAG production per cell over time, and led to increased expression of the chondrogenic markers *SOX-9*, *ACAN*, and *COL2A*.
